# Tensile Behavior, Constitutive Model, and Deformation Mechanisms of MarBN Steel at Various Temperatures and Strain Rates

**DOI:** 10.3390/ma15248745

**Published:** 2022-12-07

**Authors:** Yifan Cai, Quanyi Wang, Meng Liu, Yunqing Jiang, Tongfei Zou, Yunru Wang, Qingsong Li, Yubing Pei, Hong Zhang, Yongjie Liu, Qingyuan Wang

**Affiliations:** 1 Failure Mechanics and Engineering Disaster Prevention and Mitigation Key Laboratory of Sichuan Province, College of Architecture and Environment, Sichuan University, Chengdu 610065, China; 2 Key Laboratory of Deep Underground Science and Engineering, Ministry of Education, Sichuan University, Chengdu 610065, China; 3 State Key Laboratory of Long-Life High Temperature Materials, Dongfang Turbine Co., Ltd., Deyang 618000, China; 4 School of Architecture and Civil Engineering, Chengdu University, Chengdu 610106, China

**Keywords:** MarBN steel, tensile behavior, deformation mechanism, constitutive model, microstructure

## Abstract

To reduce harmful gas emission and improve the operational efficiency, advanced ultra-supercritical power plants put forward higher requirements on the high temperature mechanical properties of applied materials. In this paper, the tensile behavior and deformation mechanisms of MarBN steel are discussed at different strain rates (5 × 10^−3^ s^−1^, 5 × 10^−4^ s^−1^, and 5 × 10^−5^ s^−1^) under room temperature and 630 °C. The results show that the tensile behavior of the alloy is dependent on temperature and strain rate, which derived from the balance between the average dislocation velocity and dislocation density. Furthermore, observed dynamic recrystallized grains under severe deformation reveal the existence of dynamic recovery at 630 °C, which increases the elongation compared to room temperature. Finally, three typical constitutive equations are used to quantitatively describe the tensile deformation behavior of MarBN steel under different strain rates and temperatures. Meanwhile, the constitutive model of flow stress for MarBN steel is developed based on the hyperbolic sine law.

## 1. Introduction

To reduce the emission of harmful gas in coal-fired power generation plants, such as CO_2_, CH_4_, and NO_x_, the ultra-supercritical (USC) power plants were in service in the late 1950s, which can operate at 285 bar and 620 °C with efficiency in the range of 42–46% [1,2]. An increase in the operating pressure and temperature can improve the efficiency of the power plants, resulting in the decreasing emission of CO_2_. Therefore, advanced ultra-supercritical (A-USC) power plants are developed with a steam temperature above 620 °C and pressure up to 350 bar with at least 50% operating efficiency [3]. The demands for material with high fatigue strength, high thermal conductivity, and high corrosion resistance are necessary to serve in USC and A-USC power plants. The conventionally used materials include ferric and martensitic steel strengthened for creep and fatigue, nickel-based superalloy, and austenitic stainless steel, such as P91 [4,5,6] and P92 [7,8,9,10].

MarBN steel (9% CrWCoVNbNB) is low carbon martensitic steel, strengthened by boron and MX nitrides, which is designed for the stabilization of martensitic microstructure along the previous austenitic grains boundaries (PAGBs) [11], possessing a better realistic possibility than the other common 9% Cr alloys, such as P92. Therefore, the material is expected to be one of the candidate materials for A-USC power plants [12]. A variety of tensile studies on 9% Cr steel have been carried out in recent years. Choudhary et al. [13] delineated three different temperature regimes for the tensile properties of boron added reduced nitrogen containing P91 steel at different temperatures and strain rates. Chen et al. [14] performed a small punch tensile test on P91 steel, indicating that the recovered grains are elongated along the plastic flow direction and the void coalescence at the recovered grain boundaries affect the final fracture. During the tensile deformation of Cr-Mn-Si-Ni alloy steel at room temperature (RT), dislocation rearrangement occurs sequentially, and the transformation from dislocations to low angle grain boundaries (LAGBs) and high angle grain boundaries (HAGBs) leads to the refinement of microstructure [15]. Moreover, the variation in tensile properties is influenced by prior austenite grain boundaries (PAGBs), precipitation size, and enhanced recovery process [16,17]. Wang et al. [18] quantified the effect of pre-fatigue damage on the residual tensile properties of P92 steel. Over a wide range of temperatures and strain rates, the flow stress and work-hardening behavior of P92 steel can be adequately described by the Voce equation [19]. Zhang et al. [20] proposes a modified Johnson–Cook model for the tensile deformation of 9% Cr steel under the prior fatigue loading. Recently, many researchers have investigated the failure mechanism of MarBN steel through low cycle fatigue (LCF) and creep tests at RT and high temperatures [21,22,23,24]. However, the material behavior under dynamic loading differs from that under static loading. Until now, limited investigations have been performed on the tensile behavior and deformation mechanisms under different strain rates at RT and high temperatures.

Therefore, in the current study, tensile tests were carried out under different temperatures and strain rates. The deformation mechanism and fracture characteristics of MarBN steel were analyzed comprehensively. In addition, a constitutive model of flow stress for MarBN steel is developed based on the hyperbolic sine law.

## 2. Experimental Procedures

MarBN steel is used in the present work. The detailed chemical compositions of the selected alloy are listed in Table 1. The cylindrical tensile samples with the gauge diameter of 6 mm [25] and 5 mm [21] were used during standard uniaxial tensile tests at room [26] and high-temperature [27], respectively. Before the tests, the surface of the gauge section was polished mechanically with different emery paper to reduce the impact of machining process. Finally, the surface roughness reaches 0.2–0.4 μm. The RT and high-temperature tensile tests were performed at various strain rates, i.e., 5 × 10^−3^ s^−1^, 5 × 10^−4^ s^−1^, and 5 × 10^−5^ s^−1^. Before high temperature tensile tests, the specimens were heated to 630 °C and held for 20 min to obtain a balanced temperature distribution along specimen. Three specimens were employed at each temperature to reduce the data scatter.

Before tests, the specimen was observed by electron backscattered diffraction (EBSD) and transmission electron microscopy (TEM) to address the microstructure characterization. After tests, the fracture surface at each temperature was observed by scanning electron microscope (SEM) to explore the microstructure and fracture patterns, deducing the micro-damage mechanism at different temperatures and strain rates.

## 3. Results and Discussion

### 3.1. Initial Microstructure Characterizations

**Figure 1:**
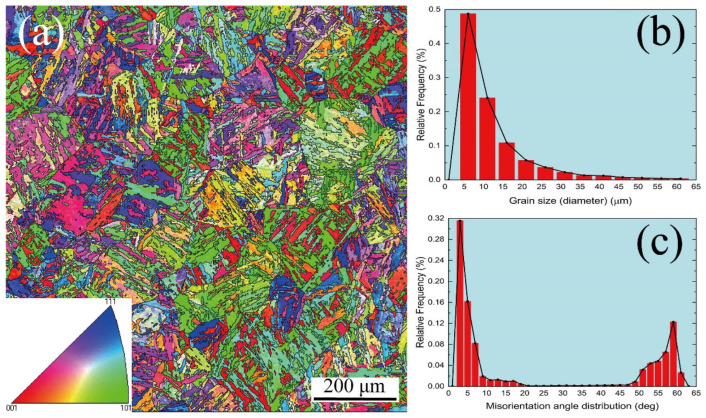
EBSD image of the as-received MarBN steel: (**a**) polo figures; (**b**) grain size distribution; (**c**) misorientation angle distribution.

Figure 1 shows the EBSD microstructure characterization of as-received MarBN steel. From Figure 1a, different phases can be observed, such as austenitic grains presented with green color and parallel martensite laths described with red color. In addition, the grain size and misorientation angle distribution are presented in Figure 1b,c, where the minimum and maximum grain sizes are about 5 μm and 63 μm, respectively, and average grain size is about 13 μm; the misorientation angle is mainly distributed in 0–20 degrees and 50–65 degrees, and average misorientation angle is about 24 degrees.

**Figure 2:**
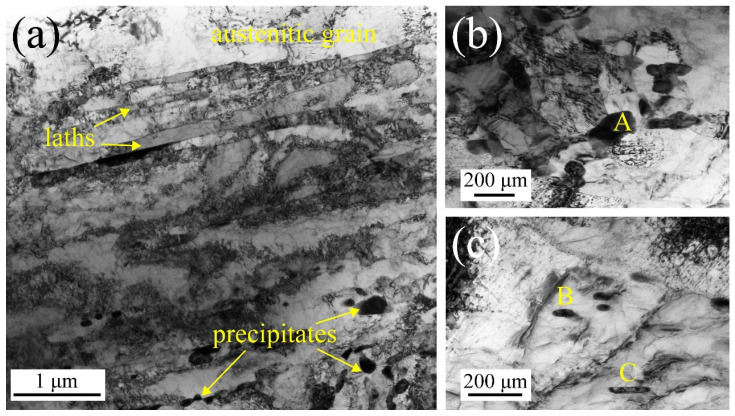
TEM image of the as-received MarBN steel: (**a**) martensite laths with high dislocation density; (**b**,**c**) different types of precipitates.

TEM is also performed to understand the microstructure more clearly. As shown in Figure 2, parallel martensitic laths with high dislocation density and a few precipitates along the grain boundary and within the grain are observed. The chemical compositions of selected precipitates along the grain boundary (Figure 2b) and within grain (Figure 2c) are presented in Table 2 with EDS analysis. The precipitates A and B with ellipsoidal shapes are mainly composed of Fe and Cr elements, resulting in Fe-Cr-rich MX carbides. The precipitate C with rectangle shape is mainly composed of Fe, resulting in Fe-rich M_3_C carbides. Therefore, two types of precipitates are presented in the as-received MarBN steel.

### 3.2. Dependence on Temperatures and Strain Rates of Tensile Behavior

**Figure 3:**
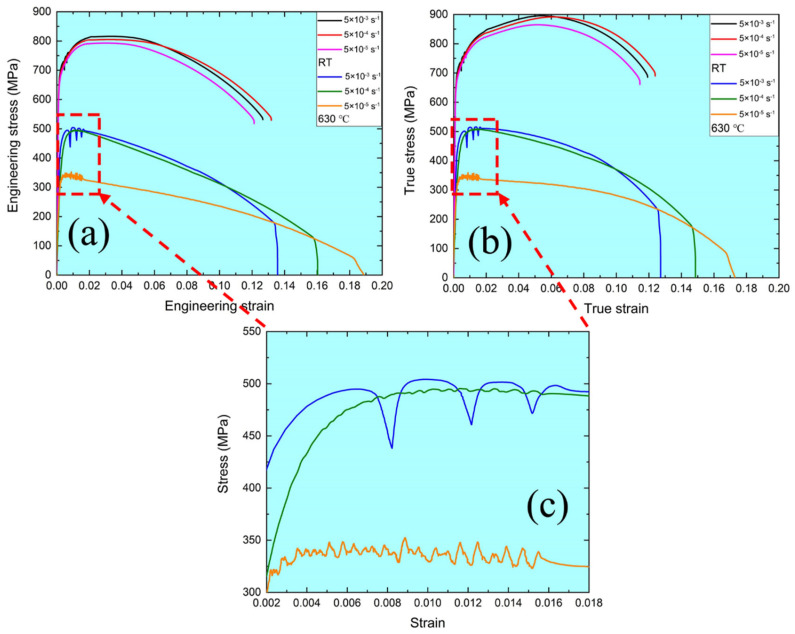
Stress-strain curves of tensile samples under different strain rates at RT and 630 °C: (**a**) engineering stress-strain curves; (**b**) true stress-strain curves; (**c**) the magnified image for the red dotted rectangle in Figure 3a,b.

Figure 3 presents the stress–strain curves under different strain rates at RT and 630 °C, such as true and engineering stress–strain curves, respectively. Clearly, the flow stress is sensitive to the strain rate and temperature under both stress–strain curves. The higher the temperature, the lower the yield and tensile strength under both stress–strain curves. These results indicate that the softening behavior is observed at 630 °C. Under a constant temperature, the tensile behavior degenerates with the decrease of the strain rate. However, the downtrend of tensile behavior at 630 °C is more severe than that at RT. In contrast, the elongation at 630 °C is better than that at RT. These results can be explained by the evolution of microstructure features during tensile tests at RT and 630 °C, which is analyzed in Section 3.3 in detail. In addition, the stress–strain curves at RT and 630 °C are divided into three stages. Under RT, in stage I, the stress increases with an increase in the strain below the yield strength. Stage Ⅱ is defined between the yield and tensile strength described by a long, stable hardening behavior due to the multiplication of dislocations [28,29]. The necking behavior is active after the yield strength due to the balance of the plastic deformation. Therefore, the stress increases quickly until the tensile strength is reached. In stage Ⅲ, exceeding the tensile strength, the hardening behavior transfers to the softening behavior, resulting from the severe necking until failure. Although the results of stage Ⅰ and Ⅲ are similar at RT and 630 °C, there is a significant difference in stage Ⅱ, such as hardening behavior at RT and softening behavior at 630 °C. For Figure 3, Stage Ⅱ under both stress–strain curves at 630 °C is characterized by a long and stable softening behavior. In addition, the effect of strain rate on the tensile behavior at 630 °C is more significant than that at RT, especially at the strain rate of 5 × 10^−5^ s^−1^. Notably, the serrated flow is observed under different strain rates at 630 °C, as shown in Figure 3c, which is the magnified image for the red dotted rectangle in Figure 3a,b, resulting from the dynamic strain aging (DSA) in MarBN steel at 630 °C caused by the unpinning and repining of interstitial atoms and dislocations [30]. According to the previous references [31,32], the type B and C serrations are presented during tensile tests at 630 °C. The type C serration appears at high strain rates (5 × 10^−3^ s^−1^), resulting from the very low atomic diffusion and discontinuous dislocation pinning [32], which is in agreement with [33]. By contrast, type B presents at middle and low strain rates (5 × 10^−4^ s^−1^ and 5 × 10^−5^ s^−1^), owing to enough atomic diffusion and significant dislocation pinning. Therefore, these results prove that temperatures and strain rates are critical on the tensile behavior of the MarBN steel.

**Figure 4:**
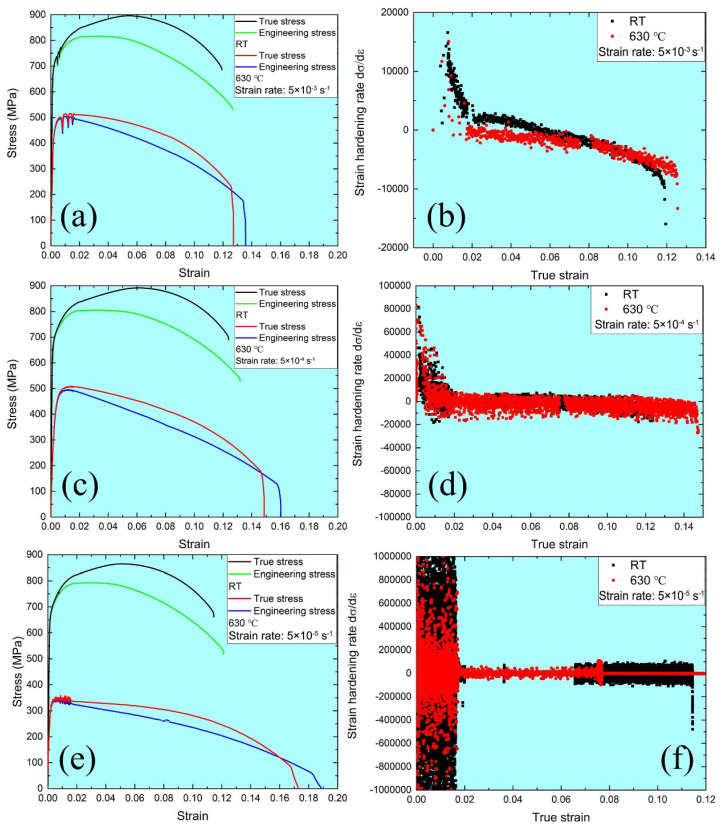
Stress–strain curves and strain hardening rate of tensile samples at RT and 630 °C: (**a**,**b**) 5 × 10^−3^ s^−1^; (**c**,**d**) 5 × 10^−4^ s^−1^; (**e**,**f**) 5 × 10^−5^ s^−1^.

To evaluate the strain hardening behavior of MarBN steel at different temperatures and strain rates, the Hollomon and Ludwik relationships [34] are used to calculate the strain hardening exponents, i.e., $n_{1}$ and $n_{2}$, which are listed in Table 3:

(1) $$\sigma \;=\;K_{1}{\varepsilon_{p}}^{n_{1}}$$

(2) $$\sigma \;=\;\sigma_{y}\;+\;K_{2}{\varepsilon_{p}}^{n_{2}}$$

where σ and $\sigma_{y}$ are the flow stress and the yield strength, respectively; $K_{1}$ and $K_{2}$ are strength parameters; $n_{1}$. and $n_{2}$ are strain hardening exponents. For both $n_{1}$ and $n_{2}$, the strain hardening capacity decreases with increasing temperature or decreasing strain rate. This result provides preliminary evidence that the strain hardening behavior of the material is sensitive to temperature and strain rate. In addition, Figure 4 shows the stress–strain curves and strain hardening rate of tensile samples at RT and 630 °C, respectively. For each strain rate, the yield and tensile strength of MarBN steel at 630 °C are lower than that at RT. The higher the temperature reaches, the faster the atomic diffuses, thus leading to the dislocations climbing and easily passing the precipitates. This process is defined as reducing the strengthening of precipitation [26], which is responsible for weakening the tensile behavior at 630 °C. Furthermore, the subgrain coarsening and decreased dislocation density [35] of MarBN steel at 630 °C are critical in controlling tensile strength at high temperatures. More detailed tensile data under different strain rates at RT and 630 °C are presented in Table 3. In addition, the clear softening behavior at 630 °C is presented under each strain rate, as shown in Figure 4. To compare and understand the softening behavior at RT and 630 °C, respectively, the strain hardening rate (dσ/dε) versus true strain curves at each strain rate are presented in Figure 4b,d,f. The value of strain hardening rate becomes zero with increasing in the true strain and partially reaches a negative value, which indicates that the dynamic recovery of recrystallization (DRX) appeared [36], and softening behavior is presented during tensile tests. For Figure 4b, at the strain rate of 5 × 10^−3^ s^−1^, the downtrend of the strain hardening rate at 630 °C is faster than that at RT. In contrast, the downtrend of the strain hardening rate at a strain rate of 5 × 10^−4^ s^−1^ is slightly different at RT and 630 °C. When the strain rate arrives at 5 × 10^−5^ s^−1^, the curves of the strain hardening rate are the same. These exciting results indicate that strain rate effect on the softening behavior is critical at RT and 630 °C. To further understand the tensile behavior under different strain rates at RT and 630 °C, Orowan’s equation is employed to consider the relationship between the strain rate and dislocation parameters [37] as follows:

(3) $$\dot{\varepsilon }\;=\;\alpha b\rho_{m}\bar{\nu }$$

where α is the orientation factor of the alloy; b is the Burgers vector; $\rho_{m}$ is the dislocation density; and $\bar{v}$ is the average velocity of dislocations, which is related to the resolved shear stress [38] as follows:

(4) $$\bar{\nu }\;=\;A\tau^{m}$$

where A and m are related to the material properties; τ is the resolved shear stress. According to Equations (3) and (4), with the increase in the strain rate, the average velocity of dislocations elevates stress. Therefore, the higher the strain rate is applied, the better the tensile behavior performs, as shown in Figure 4. The results agree with the other alloys [34] at RT. According to the previous investigations [35,39], the dislocation density and the size of a grain of MarBN steel decrease and increase at 630 °C, respectively. Therefore, the tensile strength at 630 °C is the balance between the density of dislocations affected by temperature and the average velocity of dislocations related to the strain rate. Moreover, the strain rate sensitivity at 630 °C is more significant than that at RT. Therefore, the tensile performance of MarBN steel is dependent on the strain rate and temperature, resulting from the balance between the average velocity of dislocations and the density of dislocations.

### 3.3. Fracture Pattern and Deformation Microstructure

**Figure 5:**
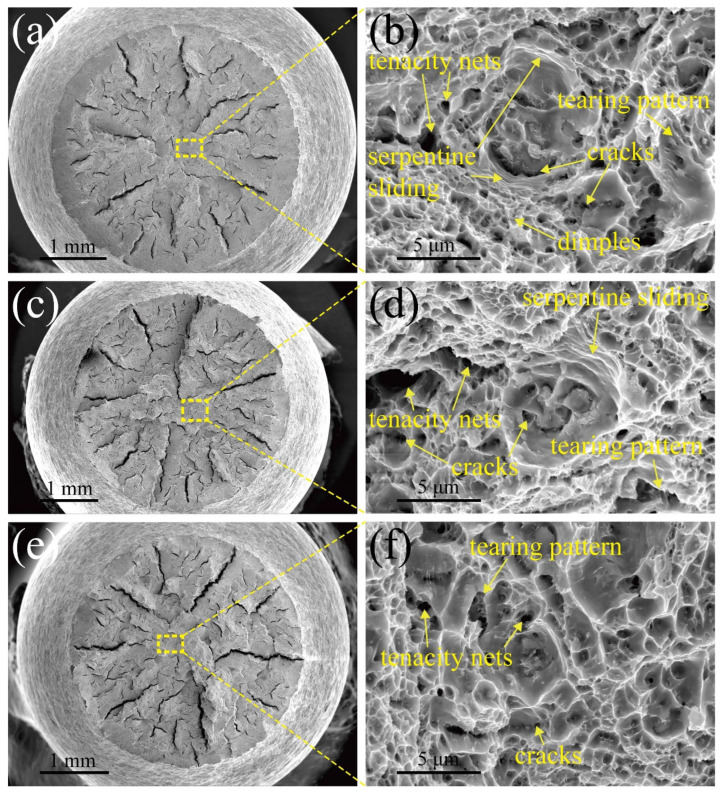
Fracture morphologies of tensile samples at RT: (**a**,**b**) 5 × 10^−3^ s^−1^; (**c**,**d**) 5 × 10^−4^ s^−1^; (**e**,**f**) 5 × 10^−5^ s^−1^.

**Figure 6:**
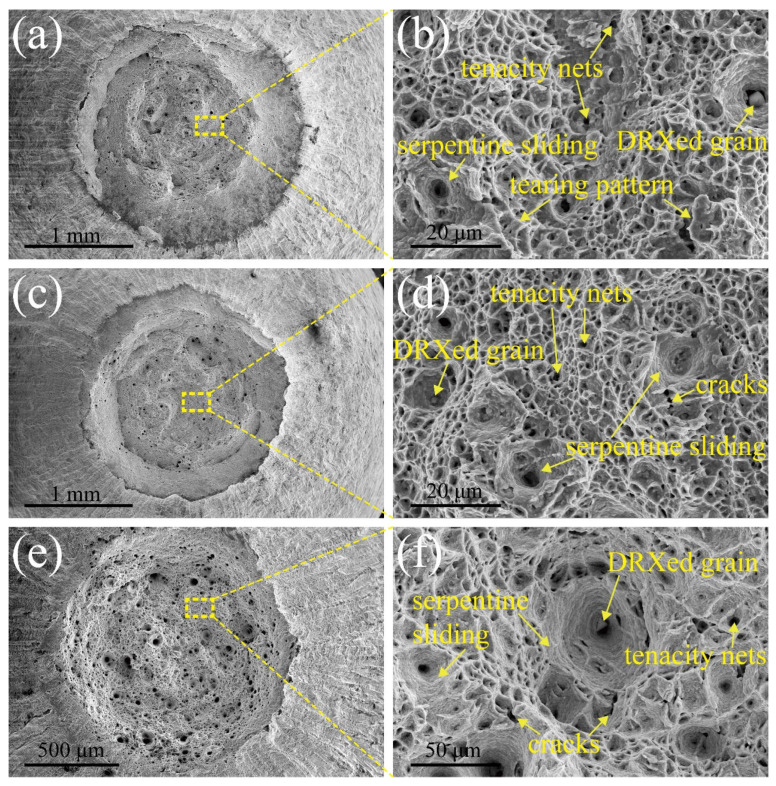
Fracture morphologies of tensile samples at 630 °C: (**a**,**b**) 5 × 10^−3^ s^−1^; (**c**,**d**) 5 × 10^−4^ s^−1^; (**e**,**f**) 5 × 10^−5^ s^−1^.

To understand the fracture mechanism of MarBN steel after tensile deformation under different strain rates, such as 5 × 10^−3^ s^−1^, 5 × 10^−4^ s^−1^, and 5 × 10^−5^ s^−1^ at RT and 630 °C, the fracture characterization was observed with SEM. Figure 5 and Figure 6 show the fracture patterns of tensile samples under different strain rates at RT and 630 °C. The cone-shape cross-section of fracture surfaces indicates the necking features due to the ductile fracture. The diameters of fracture surface after tensile tests under the strain rates of 5 × 10^−3^ s^−1^, 5 × 10^−4^ s^−1^, and 5 × 10^−5^ s^−1^ and 630 °C are measured as 3750 μm, 3650 μm, 3320 μm at RT, and 2074 μm, 1893 μm, 1250 μm at 630 °C, respectively, which indicates the diameter of fracture surface increases with elevating strain rate. To put it simply, the capacity of the necking of MarBN steel at RT and 630 °C increases with the decrease of the strain rates, which is inversely proportional to strain hardening exponents, as shown in Table 3 due to the decreasing in strain hardening capacity of MarBN steel. This result has also been verified by other alloys [40,41]. In addition, the diameters of fracture surface after tensile deformation at 630 °C are less than that at RT, which indicates that the necking behavior at 630 °C is more severe than that at RT, caused by the softening behavior with the lower strain hardening exponents of MarBN steel at 630 °C. These results agree with the tensile behavior at RT and 630 °C, as shown in Figure 3 and Figure 4 and Table 3.

Figure 5a presents the fracture surface under a strain rate of 5 × 10^−3^ s^−1^ at RT, where Figure 5b is an enlarged view of the yellow dotted rectangle. The fracture surface mainly contains numerous dimples. Serpentine sliding and tenacity nets are presented along the wall of large dimples and in the middle of dimples due to plastic deformation [42]. The tearing patterns and cracks between the dimples are also observed. Consequently, when tensile tests are conducted under the high strain rate of 5 × 10^−3^ s^−1^ at RT, the necking behavior is active above the yield strength, and then the dimples are initiated. However, the short time provided by the high strain rate tensile tests is insufficient to grow dimples, which blocks the movement of dislocation and grain boundary during plastic deformation and improves mechanical behavior, as shown in Figure 4. From Figure 5c,d, an enlarged view of the yellow dotted rectangle in Figure 5c under a strain rate of 5 × 10^−4^ s^−1^ at RT, serpentine sliding, and tenacity nets are also observed.

In addition, tearing features and cracks between the dimples are presented. However, the size and number of dimples increase and decrease, as the strain rate descends. It indicates that the deformation mechanism is similar to that under strain rate of 5 × 10^−3^ s^−1^. Furthermore, as the strain rate reaches 5 × 10^−5^ s^−1^, as shown in Figure 5f, where an enlarged view of the yellow dotted rectangle in Figure 5e at RT. Only tenacity nets and tearing patterns are observed. The dimple size is larger than the other strain rates; the number of dimples, by contrast, is the least and, because of that, the lowest strain rate of 5 × 10^−5^ s^−1^ provides enough time to develop dimples. In addition, the dimples become flatter. These growth and coalescence of dimples can reduce the plastic deformation capacity. The evidence is presented in Figure 4.

Figure 6a presents the fracture surface under the strain rate of 5 × 10^−3^ s^−1^ at 630 °C, where Figure 6b is an enlarged view of the yellow dotted rectangle. The microstructure features are similar to the results at RT, such as tenacity nets, tearing pattern, and serpentine sliding along the wall of the dimples. However, a small particle at the bottom of the dimple is defined as dynamic recrystallized (DRXed) grain caused by the dynamic recovery and recrystallization behavior under the strain rate of 5 × 10^−3^ s^−1^ at 630 °C, resulting from the severe deformation during the tensile loading condition. The formation of DRXed grain releases the stress caused by the tensile deformation and decreases the capacity of the grain boundary sliding, leading to improve the elongation of MarBN steel during the high temperature tensile loading condition. Thus, this is the reason to explain the good elongation during tensile tests at 630 °C, as shown in Figure 3 and Figure 4. The strain rate reaches 5 × 10^−4^ s^−1^ and 5 × 10^−5^ s^−1^, as shown in Figure 6c–f, respectively. The microstructure patterns of fracture surfaces are similar to the results under the high strain rate of 5 × 10^−3^ s^−1^ at 630 °C, i.e., serpentine sliding, tenacity nets, and DRXed grain. Moreover, the cracks between the middle of dimples are also presented.

In addition, with the decrease of the strain rate at 630 °C, the size of dimples increases while the number of dimples decreases, resulting from enough time to grow up under a low strain rate. Therefore, DRXed grains are observed under all the tensile loading conditions at 630 °C, implying that the temperature is the critical factor to activate the DRX behavior of MarBN steel during tensile tests.

### 3.4. Constitutive Models

To understand the effect of plastic deformation parameters on tensile properties of MarBN steel, the constitutive model, i.e., the Arrhenius-type phenomenological (AP) model [43,44], can be used to fit the true stress–strain curves, as shown in Figure 3 and Figure 4, as follows:

(5) $$\dot{\varepsilon }\;=\;A_{1}\sigma^{n^{\prime }}\exp\left(\frac{-Q_{1}}{RT}\right)$$

(6) $$\dot{\varepsilon }\;=\;A_{2}\exp\left(\beta \sigma \right)\exp\left(\frac{-Q_{2}}{RT}\right)$$

(7) $$\dot{\varepsilon }\;=\;A_{3}{\left[\sinh\left(\alpha \sigma \right)\right]}^{n}\exp\left(\frac{-Q_{3}}{RT}\right)$$

where $\dot{\varepsilon }$ is the strain rate, s^−1^; σ is the true stress, MPa; $Q_{1}$, $Q_{2}$, $Q_{3}$ are the tensile deformation activation energy, kJ∙mol^−1^; T is the tensile temperature, K; R is the gas constant and defined as the 8.31 J∙mol^−1^∙K^−1^; n and n′ are the stress exponents; $A_{1}$, $A_{2}$, $A_{3}$, β and α are the material constants, here α = β/n′. Equation (5) is fitted to the low stresses, such as ασ < 0.8. Under the high stresses ασ > 1.2, the true stress is presented by Equation (6). It should be pointed out that Equation (7) can be predicted the stress for all kinds of strain rates and temperatures.

To determinate the material constants under different strain rates at RT and 630 °C, Equations (5)–(7) can be arranged by natural logarithms on both sides as follows:

(8) $$\ln\dot{\varepsilon }\;=\;\ln A_{1}\;+\;n^{\prime }\ln\sigma \;-\;\frac{Q_{1}}{RT}\Rightarrow \ln\sigma \;=\;\frac{\ln\dot{\varepsilon }\;+\;\frac{Q_{1}}{RT}\;-\;\ln A_{1}}{n^{\prime }}$$

(9) $$\ln\dot{\varepsilon }\;=\;\ln A_{2}\;+\;\beta \sigma \;-\;\frac{Q_{2}}{RT}\Rightarrow \sigma \;=\;\frac{\ln\dot{\varepsilon }\;+\;\frac{Q_{2}}{RT}\;-\;{\mathrm{lnA}}_{2}}{\beta }$$

(10) $$\ln\dot{\varepsilon }\;=\;\ln A_{3}\;+\;n\ln\left[\sinh\left(\alpha \sigma \right)\right]\;-\;\frac{Q_{3}}{RT}\Rightarrow \ln\left[\sinh\left(\alpha \sigma \right)\right]\;=\;\frac{Q_{3}}{nRT}\;+\;\frac{\ln\dot{\varepsilon }\;-\;\ln A_{3}}{n}$$

**Figure 7:**
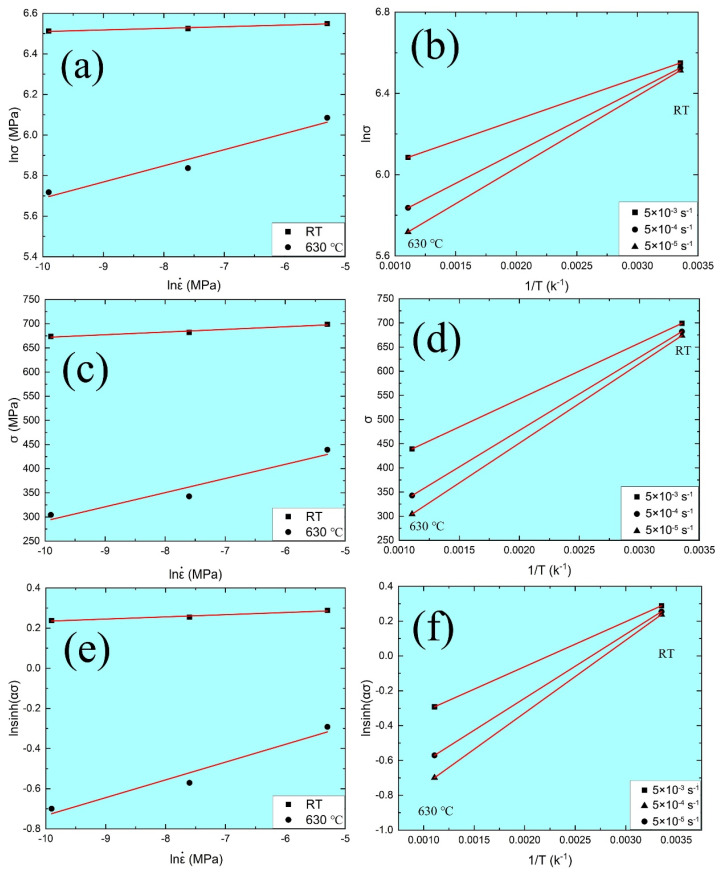
Relationships among different parameters: (**a**) lnσ − $ln\dot{\varepsilon }$; (**b**) lnσ − 1/*T*; (**c**) σ − $ln\dot{\varepsilon }$; (**d**) σ − 1/*T*; (**e**) $lnsinh\left(\alpha \sigma \right)$ − $ln\dot{\varepsilon }$; (**f**) $lnsinh\left(\alpha \sigma \right)$ − 1/*T*.

From Equations (8)–(10), the relationships among the $\ln\sigma \;-\;\ln\dot{\varepsilon }$, lnσ − 1/T, $\sigma \;-\;\ln\dot{\varepsilon }$, σ − 1/T, $\ln\sinh(\alpha \sigma )\;-\;\ln\dot{\varepsilon }$, and lnsinh(ασ) − 1/T are presented in Figure 7 under different strain rates at the strain of 0.2%, respectively, where the material parameters of $n^{\prime }$, β, n, Q can be fitted, as shown in Table 4. The deformation activation energy presents the threshold energy of the atomic transition during the tensile deformation [41], where the value in the current investigation is 128.988–164.983 kJ∙mol^−1^ under different strain rates at RT and 630 °C.

**Figure 8:**
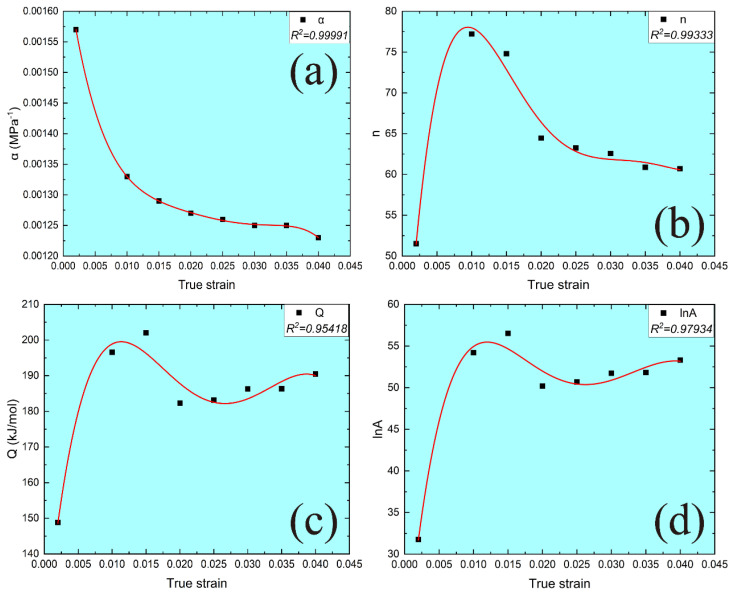
Relationships between true strain and different material parameters at different true strains: (**a**) α; (**b**) n; (**c**) Q; (**d**) lnA.

In addition, according to the previous references [45,46], the deformation activation energy and other material parameters presented in Equation (10) are related to the strain during tensile tests. The material parameters (n, Q, lnA, α) under different strains are evaluated by Equation (7) for all the stress levels, as shown in Table 5. The polynomial function is constituted to evaluate the relationships between true strain and material parameters under different true strains. The polynomial order is fitted from 2 to 9, and then a five-order polynomial is used to provide the best accuracy and correlation, as shown in Figure 8. The five-order polynomial model between true stress and material parameters is presented as follows:

(11) $$\left[\begin{array}{c} \alpha \\ n\\ Q\\ \ln A \end{array}\right]\;=\;\left[\begin{array}{cccccc} 0.00171 & -0.07916 & 6.27302 & -261.01562 & 5441.16657 & -44628.7807\\ 28.98719 & 13675.41086 & -1324836.85838 & 5.41253 & -1.01291 & 7.16162\\ 115.80016 & 19256.03859 & -1512905.04123 & 4.75582e7 & -5.89332e8 & 1.85288e9\\ 16.62556 & 8876.5196 & -717586.98648 & 2.49357e7 & -3.83345e8 & 2.10068e9 \end{array}\right]\left[\begin{array}{c} 1\\ \varepsilon \\ \varepsilon^{2}\\ \varepsilon^{3}\\ \varepsilon^{4}\\ \varepsilon^{5} \end{array}\right]$$

The effect of the strain rates and temperatures on the tensile deformation behavior can be presented by the Zener–Hollomon parameter (Z) [43,44]. Therefore, coupling Equations (7) and (12), the AP model of MarBN steel under different strain rates and temperature can be constituted as follows:

(12) $$\left\{\begin{array}{l} \left[\begin{array}{c} \alpha \\ n\\ Q\\ \ln A \end{array}\right]\;=\;\left[\begin{array}{cccccc} 0.00171 & -0.07916 & 6.27302 & -261.01562 & 5441.16657 & -44628.7807\\ 28.98719 & 13675.41086 & -1324836.85838 & 5.41253 & -1.01291 & 7.16162\\ 115.80016 & 19256.03859 & -1512905.04123 & 4.75582e7 & -5.89332e8 & 1.85288e9\\ 16.62556 & 8876.5196 & -717586.98648 & 2.49357e7 & -3.83345e8 & 2.10068e9 \end{array}\right]\left[\begin{array}{c} 1\\ \varepsilon \\ \varepsilon^{2}\\ \varepsilon^{3}\\ \varepsilon^{4}\\ \varepsilon^{5} \end{array}\right]\\ Z\;=\;\dot{\varepsilon }\exp\left(\frac{Q}{RT}\right)\\ \sigma \;=\;\frac{1}{\alpha }ar\sinh\left({\left(\frac{Z}{A}\right)}^{1/n}\right) \end{array}\right.$$

## 4. Conclusions

The tensile behavior of MarBN steel under different strain rates at RT and 630 °C were investigated in the present work. The temperature and strain rate dependence on the microstructure and deformation mechanism were observed and studied systematically. Several conclusions can be drawn as follows:

(1) MarBN steel has a martensitic lath structure with an average grain size of about 13 μm, including Fe-Cr-rich MX carbides along with the martensitic laths and Fe-rich M_3_C carbides within PAGBs.
(2) The flow stress curves present two modes, i.e., hardening behavior at RT and softening behavior at 630 °C from yield point to ultimate tensile strength. The tensile behavior is sensitive to the strain rate, especially at 630 °C, which is the balance between the average dislocation velocity and dislocation density. In addition, the serrated flow is observed under different strain rates at 630 °C due to the dynamic strain aging behavior.
(3) The deformation mechanisms are also related to the temperatures and strain rates. Apart from the presence of tenacity nets, tearing pattern, and serpentine sliding along the wall of the dimples at RT and 630 °C, DRXed grains are solely observed at high temperature which improves the elongation of MarBN steel. Combined with the strain hardening rate curves, dimples at low strain rates are large in size and small in number, reducing the plastic deformation capacity, which is due to the growth and coalescence of dimples benefit from sufficient responding time.
(4) Three typical constitutive equations are employed to quantitatively describe the tensile deformation behavior of MarBN steel under different strain rates at RT and 630 °C. A fifth-order polynomial is used to fit the material constants. After that, the constitutive model of flow stress for MarBN steel is developed based on the hyperbolic sine law.

## Figures and Tables

**Table 1:** Detailed chemical compositions of MarBN steel (wt.%).

C	Si	Mn	Cr	Mo	Ni	W	Co	V	Fe
0.12	0.06	0.06	8.95	0.15	0.16	2.80	2.84	0.2	Bal

**Table 2:** Detailed chemical compositions of different types of precipitates (wt.%).

Precipitate	Element	C	Cr	Fe	W
A	Mass fraction	1.14	22.88	35.41	40.57
B	Mass fraction	1.79	46.97	38.09	13.15
C	Mass fraction	0.65	8.38	89.65	1.32

**Table 3:** Detailed tensile behavior under different strain rates at RT and 630 °C.

Temperature/°C	Strain Rates/s^−1^	Yield Strength/MPa	Tensile Strength/MPa	Elongation/%	$n_{1}$	$n_{2}$
RT	5 × 10^−3^	699.05	907.88	11.92	0.062	0.607
	5 × 10^−4^	651.64	891.66	12.38	0.053	0.560
	5 × 10^−5^	625.76	864.72	11.45	0.047	0.532
630	5 × 10^−3^	439.25	515.58	12.72	0.047	0.572
	5 × 10^−4^	406.18	508.46	14.87	0.011	0.528
	5 × 10^−5^	256.73	358.05	17.26	0.004	0.342

**Table 4:** Detailed fitted parameters of AP model under different strain rates at RT and 630 °C.

Parameters	$n^{\prime }$	β	n	$Q_{1}$	$Q_{2}$	$Q_{1}$	α
Average-value	68.700	0.108	51.523	164.983	128.988	148.833	0.00157

**Table 5:** Detailed fitted material parameters at different true strains.

True Strain	α	n	Q	lnA
0.002	0.00157	51.523	148.833	31.768
0.01	0.00133	77.211	196.589	54.205
0.015	0.00129	74.807	202.045	56.527
0.02	0.00127	64.452	182.299	50.189
0.025	0.00126	63.257	183.151	50.689
0.03	0.00125	62.577	186.303	51.741
0.035	0.00125	60.877	186.336	51.821
0.04	0.00123	60.710	190.491	53.315

## Data Availability

The data that support the findings of this study are available from the corresponding author upon reasonable request.
